# The Stria Vascularis: Renewed Attention on a Key Player in Age-Related Hearing Loss

**DOI:** 10.3390/ijms25105391

**Published:** 2024-05-15

**Authors:** Sonny Bovee, Georg M. Klump, Christine Köppl, Sonja J. Pyott

**Affiliations:** 1 Department of Neuroscience, School of Medicine and Health Science, Carl von Ossietzky Universität Oldenburg, 26129 Oldenburg, Germany; sonny.bovee@uol.de (S.B.); georg.klump@uni-oldenburg.de (G.M.K.); christine.koeppl@uol.de (C.K.); 2 Cluster of Excellence “Hearing4all”, Carl von Ossietzky Universität Oldenburg, 26129 Oldenburg, Germany; 3 Research Centre Neurosensory Science, Carl von Ossietzky Universität Oldenburg, 26129 Oldenburg, Germany; 4 Department of Otorhinolaryngology/Head and Neck Surgery, University Medical Center Groningen, University of Groningen, P.O. Box 30.001, 9700 RB Groningen, The Netherlands; 5 The Research School of Behavioural and Cognitive Neurosciences, University of Groningen, P.O. Box 30.001, 9700 RB Groningen, The Netherlands

**Keywords:** hearing, auditory, cochlea, metabolic presbyacusis, age-related hearing loss, stria vascularis, endocochlear potential, ion transport, animal models

## Abstract

Age-related hearing loss (HL), or presbycusis, is a complex and heterogeneous condition, affecting a significant portion of older adults and involving various interacting mechanisms. Metabolic presbycusis, a type of age-related HL, is characterized by the dysfunction of the stria vascularis, which is crucial for maintaining the endocochlear potential necessary for hearing. Although attention on metabolic presbycusis has waned in recent years, research continues to identify strial pathology as a key factor in age-related HL. This narrative review integrates past and recent research, bridging findings from animal models and human studies, to examine the contributions of the stria vascularis to age-related HL. It provides a brief overview of the structure and function of the stria vascularis and then examines mechanisms contributing to age-related strial dysfunction, including altered ion transport, changes in pigmentation, inflammatory responses, and vascular atrophy. Importantly, this review outlines the contribution of metabolic mechanisms to age-related HL, highlighting areas for future research. It emphasizes the complex interdependence of metabolic and sensorineural mechanisms in the pathology of age-related HL and highlights the importance of animal models in understanding the underlying mechanisms. The comprehensive and mechanistic investigation of all factors contributing to age-related HL, including cochlear metabolic dysfunction, remains crucial to identifying the underlying mechanisms and developing personalized, protective, and restorative treatments.

## 1. Introduction

Globally, more than 1.5 billion people currently suffer from some form of hearing loss (HL). The prevalence of HL of this severity increases exponentially with age, from 15.4% in people in their sixties to 58.2% in people above the age of ninety [1]. Given the increasing lifespan, the number individuals with HL is projected to increase to 2.5 billion (or 1 in 4) by 2050. Of those, nearly 700 million are predicted to have moderate to higher levels of HL [1]. Age-related HL, or presbycusis, has been classically subdivided by Schuknecht into four types based on human histopathological findings [2,3]. One type, termed metabolic presbycusis, relates to the dysfunction and atrophy of the stria vascularis (SV) [4,5]. The SV is a complex epithelial structure lining the lateral wall of the cochlea and plays an essential role in maintaining ion and fluid homeostasis in the cochlea [6]. Strial atrophy is usually detected as a loss in the cross-sectional area of the stria vascularis [7,8]. In humans, especially over the age of 60 years, the atrophy of the SV is observed frequently and is most severe in the apical and extreme basal regions of the cochlea [9]. By transporting potassium ions (K^+^) into the endolymph, the SV is essential for generating and maintaining the endocochlear potential (EP) of about +80 mV [10]. The EP provides the electrochemical gradient necessary for the influx of K^+^ and calcium (Ca^2+^) ions from the endolymph into the sensory hair cells via mechanoelectrical transduction channels located at the top of the hair cell stereocilia. The EP, and thus SV, is therefore necessary for mechanotransduction [11].

In recent years, research investigating (age-related) HL has increasingly concentrated on the role of the sensory hair cells and the ribbon synapses that form connections between the sensory hair cells and the spiral ganglion neurons, the primary auditory neurons [12]. Consequently, the interest in metabolic HL has declined. However, as evidenced by Schuknecht’s earlier research and supported by recent studies [13,14], age-related HL is highly heterogenous and involves various potentially interacting mechanisms. Recently a genome-wide association meta-analysis highlighted the role of the SV in human HL [15]. The SV has also been linked to Ménière’s disease, and relatively rare hereditary diseases such as Alport Syndrome, Waardenburg syndrome and Norrie disease, as well as various neglected otologic diseases (reviewed by [16,17,18]). Ménière’s disease also involves vestibular pathology, however, it is unlikely that the SV is involved directly, since the endovestibular potential is maintained independently of the cochlea [19]. Given the complexity of age-related HL, the comprehensive and mechanistic investigation of all contributing factors, including cochlear metabolic dysfunction, remains crucial to identifying the underlying mechanisms and developing personalized, protective, and restorative treatments [20,21].

In turn, comprehensive and mechanistic investigation requires research using animal models due to several limitations in investigating presbycusis directly in humans. Firstly, age-related HL is compounded by environmental factors, especially noise exposure. In humans, accurately determining lifetime noise exposure is challenging, making it difficult to disentangle noise-induced from ageing-specific pathology. Secondly, in vivo measurements beyond standard audiological assessments are difficult due to the location of the cochlea within the dense temporal bone and, therefore, tissue samples are necessary for more invasive investigation of the cochlea. The quality and quantity of donated cochlear tissue is, however, extremely limited and often insufficient for mechanistic investigation. Finally, it is very often not ethically permissible to implement invasive experimental manipulations. These various limitations can be largely overcome when using animal models, which can be studied in a controlled environment and experimentally manipulated and, therefore, provide more accurate data on the direct causes of age-related HL.

Key animal models in presbycusis research are the Mongolian gerbil (*Meriones unguiculatus*) as well as several different mouse strains, such as BALB/cJ, C57BL/6J, CBA/CaJ and CBA/J [22]. In gerbils, in contrast to mice, genetic manipulation is not readily available. However, in contrast to mice, gerbils have sensitive hearing that extends to lower frequency ranges comparable to humans [23,24]. Studying animal models, particularly the gerbil, has proven valuable in distinguishing between metabolic and sensory HL. When gerbils age while exposed to continuous noise, they exhibit variable hair cell loss, specifically affecting the outer hair cells [25]. In contrast, quiet-aged gerbils, with minimal noise exposure throughout their lifespan, consistently show only minimal inner and outer hair cell loss [25,26], in accordance with predominantly metabolic presbycusis. Synaptopathy, or the loss of auditory nerve fibers on the inner hair cells, does occur in quiet-aged gerbils [27,28,29], However, not to such an extent that it necessarily affects hearing thresholds [30,31,32]. Despite reduced acoustic exposure in quiet-aged gerbils, significant HL can still occur, providing an excellent chance to investigate the effects of strial dysfunction [33]. Furthermore, it is possible to pharmacologically reduce the EP in young gerbils using furosemide, allowing for the investigation of EP loss in the absence of other age-related effects [34,35,36].

This narrative review specifically emphasizes the critical role of the SV in age-related hearing impairment. It comprehensively integrates classical and recent studies, while also aiming to link findings from animal models with those from humans. The review begins by introducing the structure and function of the various layers of cells in the SV and the normal capillary network. Subsequently, it delves into the mechanisms contributing to the age-related loss of strial function, exploring the decline in ion transport in SV cells, alterations in pigmentation, the involvement of tissue-resident macrophages and inflammatory responses, and the role of vascular atrophy. Furthermore, this review sheds light on strial degeneration as a significant contributor to presbycusis, highlighting the value of animal models for investigating the underlying mechanisms. By focusing on the cellular and molecular processes underlying strial dysfunction, it provides insight into the causes of dysfunction and targets for potential interventions.

## 2. Structure and Function of the Stria Vascularis

**Figure 1:**
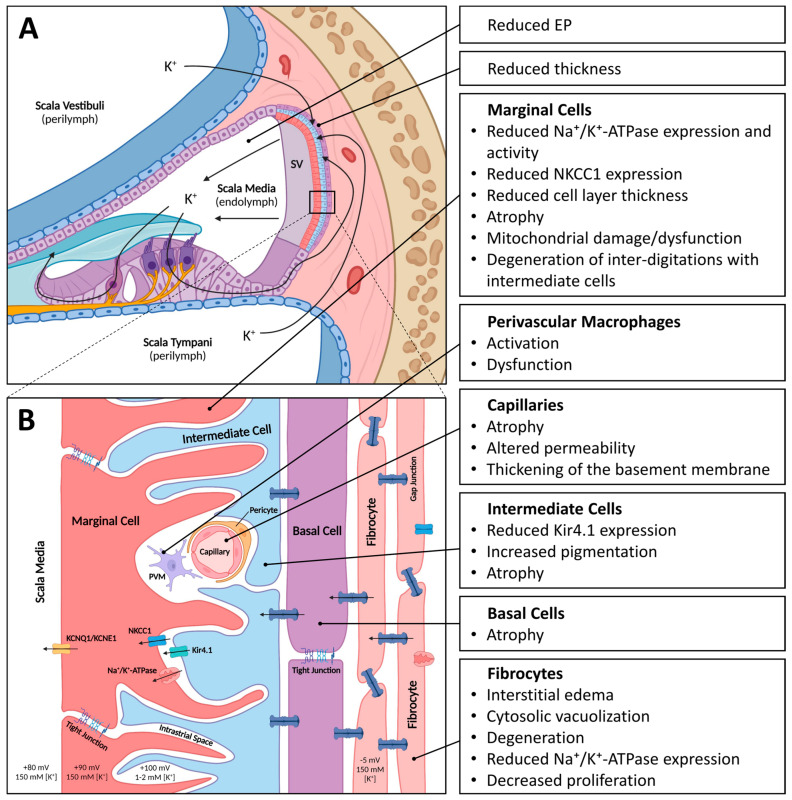
Schematic of the stria vascularis (SV) with age-related changes indicated. (**A**). Schematic cross-section of the cochlea with SV located in the lateral wall. The SV with its three main cell layers, consisting of marginal cells, intermediate cells, and basal cells, is responsible for generating and maintaining the high potassium ion (K^+^) concentration in the endolymph and resulting endocochlear potential (EP). (**B**). Magnified schematic of the cell layers of the SV. Marginal cells, connected via tight junctions, directly face the endolymph and express several ion channels and transporters (KCNQ1/KNCE1, NKCC1 and Na^+^/K^+^-ATPase). Marginal cells form extensive inter-digitations with intermediate cells, which express the ion channel Kir4.1. The intrastrial space, which has a relatively low K^+^ concentration and high positive potential, is located between the marginal and intermediate cell layers. Capillaries run through the SV and their endothelial cells, and pericytes and perivascular macrophages form the blood–labyrinth barrier (BLB). Basal cells, connected via tight junctions, form gap junctions with intermediate cells and fibrocytes to form a syncytium. Image created with BioRender.com.

The SV is a highly vascularized tissue located in the lateral wall of the cochlea. It comprises three main cell types: marginal, intermediate, and basal cells (Figure 1). In addition, an extensive network of capillaries is present within the SV, from which the name “stria vascularis” is derived. Marginal cells directly face the endolymph, intermediate cells form the middle layer, and basal cells, which are closely associated with fibrocytes in the spiral ligament, are located most laterally [37]. Together, the different cell types of the SV are responsible for K^+^ transport into the scala media and the generation of the endocochlear potential (EP). Inner hair cells, the main sensory receptors of the cochlea, as well as outer hair cells, which act as a cochlear gain control, depend on the EP for their proper functioning. The K^+^ flowing through the hair cells via their transduction and basolateral channels is then circulated back to the SV and released into the endolymph again [38]. The disturbance of this cochlear homeostasis is the cause underlying many types of HL [39]. The electrochemical details of EP generation are beyond the scope of this text and are explored in more detail elsewhere [10]. In the following sections, the functional role of each of the cell types within the SV as well as the capillary network that forms the blood labyrinth barrier are discussed.

### 2.1. Marginal Cells

Marginal cells form a monolayer epithelium that directly faces the endolymph (Figure 1). They are derived from the epithelial cells of the cochlear duct [40], and connected via tight junctions. Reflecting the importance of K^+^ transport by the SV, two key K^+^ transporters are enriched in the marginal cells of the SV: the Na^+^/K^+^-ATPase pump and the Na^+^-K^+^-2Cl^−^ cotransporter 1 [38].

The Na^+^/K^+^-ATPase pump is abundantly expressed in the basolateral membrane of marginal cells [38,41] (Figure 1B). This pump is a ubiquitously expressed transmembrane protein composed of a catalytic α subunit, a β subunit that can modulate the kinetic characteristics and acts as a chaperone during structural and functional maturation, and tissue-specific FXYD proteins that modulate activity. There are four α, four β, and seven FXYD mammalian subunit isoforms (reviewed by [42,43,44]). Na^+^/K^+^-ATPase is expressed in tissues throughout the body in various isoform combinations. The isoform combinations in a given type of tissue can also vary between species. This heterogeneity suggests that Na^+^/K^+^-ATPase adapts its activity to the tissue- and species-specific needs. The α1 isoform is the most ubiquitous isoform, while, for example, the α3 isoform is typically found in neuronal tissue. In addition to being abundantly expressed in the marginal cells of the gerbil SV (α1, β2), the Na^+^/K^+^-ATPase is also found in spiral ganglion neurons (α3, β1), including their central and peripheral processes, in subpopulations of fibrocytes (α1, α2, β1), and various other cell types [41,45]. In mice, the Na^+^/K^+^-ATPase is found in marginal cells of the SV (α1, β1, β2), subpopulations of fibrocytes (α1, α2, β1), a number of spiral ganglion neurons (α2, β1), as well as other cell types [46]. In CBA/CaJ mice, the Na^+^/K^+^-ATP α1 isoform was found to be the most abundant protein in the strial capillaries, which plays an important role in the blood–labyrinth barrier integrity [47]. In rats, the Na^+^/K^+^-ATPase α3 is expressed in spiral ganglion somata, afferent and efferent terminals, and supporting cells neighboring the inner hair cells [48]. In the human cochlea, Na^+^/K^+^-ATPase is expressed in marginal cells of the SV (α1, β1), subpopulations of fibrocytes (α1, β1), spiral ganglion neurons (α1, α3, β1), and various other cell types [49,50].

The Na^+^-K^+^-2Cl^−^ cotransporter 1, commonly known as NKCC1, is also located in the basolateral membrane of marginal cells [51]. Like the Na^+^/K^+^-ATPase pump, NKCC1 is a ubiquitously expressed transmembrane protein [52]. Unlike the Na^+^/K^+^-ATPase pump, NKCC1 is not powered by ATP but rather harnesses the Na^+^ gradient created by the Na^+^/K^+^-ATPase pump to allow the movement of two molecules of Cl^−^ and one molecule of K^+^ together with one molecule of Na^+^ into the cell [52]. In the gerbil, in addition to the marginal cells, NKCC1 is also found in subpopulations of spiral ligament fibrocytes [51]. In C57BL/6J mice, NKCC1 is present in the stria vascularis as well as in fibrocytes in the spiral ligament [53]. In humans, NKCC1 mutations are associated with HL and deafness [54].

### 2.2. Intermediate Cells

Intermediate cells are located between the marginal and basal cell layer (Figure 1). Intermediate cells show diversity in their types within and across species but commonly contribute to melanin production, protection against oxidative damage, and K^+^ transport within the SV. Intermediate cells of both melanoblast and Schwann-cell precursor origin have been found [55]. During development, they invade the lateral wall and penetrate the basement membrane beneath the marginal cells following a basal-to-apical spatiotemporal gradient [55,56,57]. The intermediate cells are also melanocytes and, like other melanocytes, produce the pigment melanin. Mammalian species have varying levels of SV pigmentation, which consists largely of melanin. No melanin is present in the albino inner ear [58]. Melanocytes are best known for their protection against UV-induced DNA damage in the skin. They produce the melanin pigment in specialized organelles called melanosomes, which are then transferred to keratinocytes [59]. Since melanin is involved in free radical scavenging [60], it likely also serves a protective role in intermediate cells. The various properties of melanin that are involved in this protective function are discussed in detail in Section 3.2.

In several species, two populations of melanin-containing cells can be identified. The two populations observed in different species are, however, not clearly equivalent and/or may represent different stages of the same cell type. In the chinchilla (*Chinchilla lanigera*), one population of melanin-containing cells are called melanocyte-like cells, which are heavily pigmented with dense pigment granules and are closely associated with blood vessels. The other population consists of intermediate cells, which contain small, dense pigment granules [61]. Similarly, in the cat, one population of melanin-containing cells are called melanocytes, which are closely associated with capillaries and contain varying numbers of fine-grained pigment granules. Intermediate cells, by contrast, are not obviously associated with capillaries and less frequently contain pigmented organelles [58]. In mice, one population of melanin containing cells are called light intermediate cells, and they are present from birth and contain pigment granules in different stages of development. Light intermediate cells appear less frequently in old animals. The other population, called dark intermediate cells, which are only present in adult mice, contain pigment granules in lysosomal bodies. There are few other organelles, and their nuclei are often pyknotic, a state characterized by the irreversible condensation of chromatin. Neither light nor dark intermediate cells are obviously associated with capillaries. These two apparently different populations may represent different stages in the life cycle of a single cell type [62].

In newborn gerbils, melanosomes in the SV are found in large, dendritic cells, called intermediate cells, while the adult gerbil SV contains a population of melanin containing cells called star-shaped, triangular, or fusiform pigment cells (STFPCs). These STFPCs correspond morphologically to the melanocyte-like cells in the chinchilla and cat, which contain most of the melanin in the adult SV. It is possible that melanosomes are transferred from intermediate cells to these STFPCs, similar to how in the dermal melanin system melanosomes are transferred to keratinocytes [63]. To a lesser extent, basal cells can also contain melanin granules, which are likely transferred from nearby melanin-producing cells [61,62]. More recently, in the gerbil, ultrastructural studies distinguished a basal subtype of intermediate cell (BIC) from an upper subtype of intermediate cell (UIC), referring to their relative positions within the strial layers. Marginal cells projected distinct processes to each of these subtypes suggesting a functional difference [64].

In addition to their role in melanin production, intermediate cells play an important role in K^+^ transport via inwardly rectifying Kir4.1 channels [65]. In the healthy adult SV, marginal cells and intermediate cells form extensive inter-digitations to maximize the surface area for ion exchange (Figure 1B) through the extension of primary and secondary processes, as was shown by ultrastructural analysis in the gerbil [66]. In the viable dominant spotting mouse mutant, which lacks intermediate cells in the SV, the development of inter-digitations between the remaining cell types is reduced and the EP remains at 0 mV, failing to show the normal gradual developmental increases to about +100 mV in adulthood. Intermediate cells are, therefore, regarded as essential for the normal development and functioning of the SV [67].

### 2.3. Basal Cells and Fibrocytes

Basal cells form the third and final layer of the SV (Figure 1). They are derived from the otic mesenchyme [68]. The basal cell layer is located next to the spiral ligament, which contains five different types of fibrocytes (I–V) bathed in perilymph. Gap junctions composed of connexins connect the basal cells to the fibrocytes and intermediate cells in the SV, forming a functional syncytium called the “connective tissue gap junction network” [38,69]. To form a barrier between the intrastrial space and the perilymph in the spiral ligament, basal cells are connected to each other via tight junctions containing the adhesion molecule claudin-11 [70].

Fibrocytes play an active role in regulating ion concentrations. Specifically, Na^+^/K^+^-ATPase and NKCC1 are abundantly expressed in type II and IV fibrocytes [41,46,51,71]. Via the gap junction network with the basal cells and intermediate cells, the K^+^ taken up by the fibrocytes is transported to the SV [69]. Together, these various cell types play essential roles in maintaining the appropriate ionic environment and fluid balance in the cochlea.

### 2.4. Capillary Network and the Blood–Labyrinth Barrier

The SV contains an extensive capillary system (Figure 1B) and, similar to the brain, which is protected by the blood–brain barrier (BBB), the cochlea is protected by the blood–labyrinth barrier (BLB). Like the BBB, the BLB restricts the entry of most bloodborne compounds. Both in the BBB and the BLB, the endothelial cells of the capillaries are connected via tight junctions, and the vessels are populated with pericytes [72,73]. The permeability and active transport mechanisms of the BLB, however, differ from those in the BBB and are not yet well characterized. The measurements of permeability using a radioactive tracer showed that the BLB is less permeable than the BBB at the choroid plexus [74]. Interestingly, the permeability appears to be molecule specific: for example, gentamycin can cross the BLB but not the BBB [75]. The restrictive entry of bloodborne compounds into the cochlea due to the BLB and the incomplete characterization of its permeability also complicates the development of drug delivery via the strial vasculature [73]. The permeability of the blood vessels is further regulated by perivascular macrophages, a subset of tissue resident macrophages [76]. Tissue resident macrophages within the cochlea can have different origins. In mice, during development, resident macrophages originating from the yolk sac as well as resident macrophages originating from the fetal liver are found [77]. Resident macrophages in the SV only appear after birth [77]. In the adult mouse cochlea, bone marrow-derived resident macrophages are found in the spiral ligament, the auditory nerve, and the SV [78,79,80]. Their function and especially role in inflammatory responses is discussed in Section 3.3.

## 3. Mechanisms Contributing to Age-Related Loss of Strial Function

To assess the impact of SV dysfunction on the EP and hearing, researchers have employed various functional and structural measures. In humans, distinguishing between the metabolic component of HL, resulting from SV dysfunction, and the sensory component, resulting from hair cell dysfunction, remains challenging because there is currently no method for assessing these tissues directly in a noninvasive and independent manner. Based on the shape of the audiogram, presbycusis has been categorized as metabolic, sensory, or both [81,82]. These categories, however, did not indicate the specific extent to which either component contributed to HL. More recently, a promising quantitative approach has been developed to estimate indirectly the extent of metabolic and sensory HL through analyzing the shape of the audiogram [14]. While it is not possible to completely define or differentiate the underlying pathologies solely based on the audiogram, the findings indicate that the audiogram provides valuable insights into both types of HL. This method was recently validated using datasets from gerbils and humans, including histopathologic assessments. The model could potentially be improved in the future by including the interactions between the two pathologies [14]. A study using human samples confirmed that a flatter audiometric shape is associated with greater strial atrophy, however, it was argued that not the flatness but the degree of low-frequency threshold shift is predictive of strial degeneration [83].

In animal models, the EP and, thus, SV function can be measured directly but invasively using microelectrodes placed in the cochlea [84,85]. These measurements detect the loss of the EP in response to various perturbations of the SV, including the absence of melanocytes or melanin [67,86], potassium ion channel knockout [87], and furosemide application [34,88]. Structural measures of the SV, used to estimate the extent of strial atrophy, commonly include measures of the SV thickness, cross-sectional area, and/or cell density [7,8,89]. These changes can be assessed postmortem in animal models as well as humans, with the caveat that histological changes in humans are often examined with a limited knowledge of the individual’s hearing history and well after the onset of HL, complicating the interpretation of the causative pathology [7,8,83]. In humans, structural measurements reveal correlations between the extent of strial atrophy and HL, supporting the basis of Schuknecht’s original classification of metabolic presbycusis [2,3,7,83]. Various correlations have also been observed in animal models. A decrease in marginal cell density, for example, was shown to be a good predictor of EP across the lifespan in BALB/cJ mice. In addition, spiral ligament thickness was also found to be a good predictor of EP across the lifespan of BALB/cJ mice [89], supporting the involvement of spiral ligament fibrocytes in age-dependent lateral wall atrophy [90]. Interestingly, the examination of CD/1 mice, which show accelerated presbycusis, suggests that the fibrocyte pathology of the SV preceded the age-related pathology of the sensorineural structures in the cochlea [91].

Nevertheless, there is considerable discrepancy between functional and structural measures of the SV, with functional deficits sometimes observed in the absence of structural pathology and vice versa. For example, in BALB/cJ mice, strial thickness was found to be only a weak predictor of the age-related loss of the EP, while in C57BL/6J mice no significant correlation was found. Moreover, other morphological changes that showed highly significant correlations with age-related changes in the EP in BALB/cJ mice, including marginal cell density and spiral ligament thickness, failed to show significant correlations in C57BL/6J mice [89]. In guinea pigs with sensorineural HL induced by kanamycin and xylazine, the EP returned to normal after 56 days despite the severe atrophy of the SV [92]. In CBA/CaJ mice showing permanent, noise-induced threshold shifts, the EP recovered despite strial edema and the degeneration of type II fibrocytes in the spiral ligament [93].

Such discrepancies may be explained by additionally considering the role of the sensory hair cells. Hair-cell loss per se does not affect the EP, as demonstrated in CBA/J mice, where the EP was maintained even after severe hair cell loss induced by kanamycin and furosemide [94]. A reduction in hair cells would nevertheless decrease the metabolic load on the SV by reducing the continuous K^+^ current flow through active hair cells [92]. Depending on the extent of hair cell loss, a normal EP could still be observed despite SV dysfunction. By analogy, the SV is often likened to a battery that powers the hair cells. Outer hair cells, and to a lesser extent inner hair cells, draw current from this battery, and a reduced load resulting from hair cell loss would allow the EP to remain relatively normal even if the SV generating the EP was substantially weakened [13]. In support of this phenomenon, C57BL/6 mice with significant outer hair cell loss showed preserved EPs, despite a decreased expression of the K^+^ channels Kir4.1 and KCNQ1 and a decreased expression of the transcripts encoding Na^+^/K^+^-ATPase α1 and α2 and NKCC1 in the SV [95]. Moreover, studies in gerbils have shown that outer hair cells can adapt their transducer operation point in response to an acutely lowered EP, recovering nearly full cochlear amplification [96]. It is not known if this adaptive mechanism also operates in the long term when the EP is chronically reduced due to aging [96]. These findings highlight the complicated interaction between the SV and the hair cells and, thus, metabolic and sensory HL.

Perhaps not surprisingly then, there is considerable controversy over the relative contributions of strial atrophy and sensorineural loss to age-related HL in humans. A recent analysis of hair cells, auditory nerve fibers, and strial tissue in human post-mortem tissue showed that most of the variance in audiometric thresholds could be explained by hair cell loss and not strial damage [8]. More recently, a related study with additional samples did find a significant predictive value of strial degeneration for threshold shift in the low frequency region, although the contribution of outer hair cell damage was still greater [83]. However, as further discussed below, strial degeneration, assessed by the common structural measures outlined above, is not necessarily a reliable indicator of the functional status of the SV. Thus, the contribution of strial function, beyond just strial degeneration, in age-related HL in humans warrants increased investigation. Therefore, the following sections will explore the various factors implicated in SV function and their potential for assessing strial dysfunction during aging. These factors include disrupted ion transport, altered pigmentation, inflammatory responses, and vascular atrophy.

### 3.1. Disrupted Ion Transport

This section summarizes how aging and experimental reduction in the expression and function of crucial K^+^ channels and transporters, specifically Na^+^/K^+^-ATPase, NKCC1, and Kir4.1 in the SV, impact the generation of the EP, strial function, and hearing thresholds.

In the quiet-aged gerbil, the expression level of the Na^+^/K^+^-ATPase, based on immunoreactivity observed in the marginal cells of the SV, has been shown to decline with age [97]. Similarly, in aged CBA/CaJ mice, Na^+^/K^+^-ATPase expression in the SV decreases by approximately 80% despite a reduction in strial thickness by only 20% [98]. This observation indicates that strial atrophy is a poor predictor of ion transport function in the SV. In the quiet aged gerbil, in 21–22-month-old animals, only a limited, patchy loss in immunoreactivity was observed in apical cochlear turns. In contrast, in 29–31-month-old gerbils, the loss of Na^+^/K^+^-ATPase immunoreactivity expanded to most of the apical turn and parts of the basal turn [97]. In the oldest group of gerbils, aged 35–38 months, the loss of immunoreactivity extended throughout the apical turn and into basal turns and showed considerable variation, involving the entire basal turn in the most severe cases. In regions of advanced strial atrophy, fibrocytes in the lateral wall also showed a decrease in Na^+^/K^+^-ATPase content [97]. In quiet-aged gerbils, aged 33–36 months, fibrocytes in the spiral ligament showed cytosolic vacuolization and degenerated cells, as well as interstitial edema. These conditions may be the result of impaired K^+^ diffusion through marginal cells [90]. Since Na^+^/K^+^-ATPase immunoreactivity is only an indirect indicator for its enzymatic activity, the enzymatic activity in the lateral wall has also been assessed directly. In some 36–39 months old gerbils, a marked decrease in Na^+^/K^+^-ATPase activity was observed compared to other age groups ranging from 1 to 19 months old [99]. Importantly, in the human cochlea, a decrease in Na^+^/K^+^-ATPase α1 in the SV has been related to presbycusis [100].

In gerbils, the proportion of the SV showing immunostaining for Na^+^/K^+^-ATPase was found to correlate with the magnitude of the EP. For evaluation purposes, the intensity of the staining was not taken into account, primarily because regions exhibiting reduced immunoreactivity, rather than complete loss of immunoreactivity, were uncommon [97]. In aged gerbils (30 months old), the decline in EP compared to young gerbils (under 8 months old) was most pronounced near both the apex and base of the cochlea [101]. Specifically, there was a decrease of 18 mV at 0.5 kHz, 3 mV at 2 kHz, 7 mV at 16 kHz, and 16 mV at 40 kHz equivalent frequency locations. In contrast, even older gerbils (36 months old) showed a more uniform decline in EP across the cochlea, with decreases of 27 mV at 0.5 kHz, 19 mV at 2 kHz, 23 mV at 16 kHz, and 31 mV at 40 kHz [101]. Furthermore, in aged gerbils (32–39 months old), there was a strong correlation between reduced Na^+^/K^+^-ATPase activity and reduced EP, suggesting that this reduced activity can explain most of the decline in EP [102]. No significant difference in endolymphatic K^+^ concentration was found in quiet-aged versus young gerbils, although the intersubject variability was much greater in aged compared to young gerbils. There was a weak but significant correlation between the K^+^ concentration in endolymph and the EP in aged gerbils (R^2^ = 0.23, *p* < 0.01). K^+^ concentrations below 150 mM were associated with EPs below 60 mV [103].

The loss of NKCC1, a Na^+^-K^+^-2Cl^−^ cotransporter located in the marginal cells of the SV and subpopulations of fibrocytes of the spiral ligament, can, just like the loss of Na^+^/K^+^-ATPase, contribute to hearing problems. In the gerbil, NKCC1 shows both a developmental increase and age-related loss of expression similar to that of Na^+^/K^+^-ATPase [71]. With age, diminished NKCC1 immunoreactivity in the SV and, eventually, along with the advanced atrophy of the SV, a complete loss of immunoreactivity was reported [71]. In the final stages of atrophy, the SV was replaced by a thin squamous cell layer [71]. In aging C57BL/6 mice, the level of NKCC1 mRNA as well as NKCC1 protein expression in the lateral wall was reduced [53]. It has been shown that mice lacking NKCC1 are deaf and the membranous labyrinth in the inner ear is collapsed [104,105]. NKCC1 has also been implicated in human HL. In three cases, deafness in children was associated with inherited mutations that led to a total absence of NKCC1. Additionally, other cases have been reported where single allele mutations in the SLC12A2 gene, which encodes NKCC1, were linked to HL. These findings linking the loss of NKCC1 function and HL in humans underscore the validity of animal models in studying the contribution of NKCC1 to metabolic HL [54].

In the gerbil, as mentioned above, NKCC1 immunostaining shows a similar developmental and age-related expression pattern to Na^+^/K^+^-ATPase, consistent with a high level of functional cooperation [71]. In Black Swiss-129/SvJ mice, heterozygote deletion of either Na^+^/K^+^-ATPase α1, Na^+^/K^+^-ATPase α2, or NKCC1 lead to progressive, age-related HL. Interestingly, when both Na^+^/K^+^-ATPase α2 (located in fibrocytes but not marginal cells) and NKCC1 (found in marginal cells and certain fibrocyte subpopulations) were simultaneously deleted, hearing remained largely preserved in Black Swiss-129/SvJ mice. This observation suggests that the dual deletion may have a protective effect on hearing. Double deletion presumably leads to downregulated but balanced K^+^ flux, potentially offering insights for future drug treatment strategies. If the downregulation or inhibition of a certain transporter due to ototoxic drugs or a genetic predisposition leads to HL, then deliberately inhibiting the counterbalancing transporter may mitigate the potential damage [106].

NKCC1 decreases with age but can also be pharmacologically manipulated. Furosemide is a loop diuretic that disturbs the function of the SV by blocking NKCC1. By applying this drug, the effects of a dysfunctional SV can be simulated in young animals, thereby allowing the evaluation of EP decline while excluding potentially confounding effects of aging. Chronic furosemide application (up to 28 days) was shown to lower the EP and induce HL, quantified using compound action potential (CAP) measurements [34]. Increased CAP thresholds were the most prominent at high frequencies, likely reflecting the greater gain of the cochlear amplifier (mediated by the outer-hair-cell function) in the high compared to low frequency regions of the cochlea [34]. Furthermore, the loss of EP and the audiogram profile resulting from furosemide treatment in young gerbils closely matched profiles of old gerbils. This observation suggests that the reduction in EP in quiet-aged gerbils is the main cause of their age-related HL [34]. A separate study revealed that when young gerbils were treated with furosemide for seven days, there was a reduction in EP and an increase in CAP thresholds [35]. Notably, two weeks after furosemide treatment was stopped, both the EP and the CAP thresholds began to recover. This recovery coincided with an observed increase in the cell division of fibrocytes within the spiral ligament. In contrast, older gerbils exhibited a decrease in fibrocyte proliferation compared to the younger controls [35]. These observations suggest that decreased fibrocyte proliferation plays a role in the pathology of the lateral wall of the cochlea, resulting in the loss of EP.

Another factor that can result in disturbed ion transport through the SV is cellular damage due to metabolic dysfunction. Since the SV has a high metabolic rate [107], it is likely highly susceptible to such a dysfunction. In 30–36-month-old gerbils, the first sign of strial atrophy was the degeneration of secondary marginal cell processes, apparently caused by oxidative self-damage to mitochondria in the primary processes, resulting in insufficient ATP for the Na^+^/K^+^-ATPase of the secondary processes. Eventually, the primary processes themselves were also affected [66]. Aging, as well as noise overexposure and ototoxic drugs, can trigger mitochondrial dysfunction resulting in oxidative stress by the excess production of reactive oxygen species (reviewed by [108,109]). Cisplatin treatment, for example, leads to an increase in free radicals within the SV, resulting in mitochondrial membrane permeabilization and the apoptosis of marginal cells [110].

The dysfunction of the K^+^ channel Kir4.1, located in the intermediate cells of the SV, contributes to HL as well. In knockout mice lacking Kir4.1, the EP is abolished [87]. The loss of Kir 4.1 protein expression in the intermediate cells of the SV is likely the direct cause of abolished EP and deafness in a mouse model of Pendred syndrome, caused by the homozygous deletion of Slc26a4 [111]. This Pendred syndrome mouse model also showed the hyperpigmentation of the SV as well as increased macrophage proliferation and activation, potentially as a result of excess free radicals [111,112]. The hyperpigmentation of the SV and tissue-resident macrophages are discussed further in Section 3.2 and Section 3.3, respectively. In aging CBA/CaJ mice, a decline in Kir4.1 immunoreactivity has been shown in intermediate cells as well as outer sulcus cells and satellite cells in the spiral ganglion [113]. The animal models of the dysfunction of Kir4.1 are highly relevant for the understanding of human age-related HL, since the expression of Kir4.1 was found to be altered in these same cell types in the aging human cochlea [113].

Collectively, these studies linking reduced EP with HL predict that artificially increasing the EP in animals suffering from age-related HL would restore hearing to some extent. This has been tested by injecting a current in the scala media of old gerbils to increase the EP, resulting in a 20 dB reduction in the CAP threshold [101]. Although this manipulation was invasive and acute, it nevertheless provides a proof of principle that restoring the EP artificially, potentially using pharmacological approaches and/or implanted biological devices, can improve hearing. Furthermore, the studies discussed above show that different kinds of dysfunction produce the same HL because the elements discussed in this section are interdependent; if any element is taken out, the whole ion transport loop suffers. This underscores the importance of differential diagnosis in this context, as the identification of the specific dysfunction or combination of dysfunctions at play allows for personalized treatment strategies targeting the root cause of the condition.

### 3.2. Variation in Pigmentation in the Stria Vascularis

This section focuses on the role that pigment plays in the SV in the context of age-related HL. As briefly mentioned in the introduction, melanin has a number of different properties that can be of importance in the SV. Certain drugs are believed to accumulate in the inner ear due to their affinity to melanin [114]. Additionally, melanin is capable of scavenging paramagnetic metal [115], consistent with its potential cytoprotective function as a scavenger of toxic substances [116]. Melanin also plays a role in free radical homeostasis and is able to scavenge and bind free radicals, which is important for combatting oxidative stress [60].

While the precise role of melanin in age-related HL is not fully understood, studies involving albino animals offer some, albeit conflicting, insights. Most notably, an investigation comparing C57BL/6J mice, which have normal melanin production, and C57BL/6J-Tyr(c-2J) mice, which carry a tyrosinase mutation resulting in the absence of melanin, found that both pigmented and albino mice exhibited similar rates of HL and sensory cell loss but that the EP in the basal cochlea was reduced in albino compared to pigmented mice. A reduced EP in aged albino mice was correlated with reduced strial thickness and a greater loss of marginal cells [86]. BALB mice, which are albino, demonstrate a significant reduction in endocochlear potential (EP) with age. Initially, albino C57BL/6J mice exhibit an aging pattern similar to their pigmented counterparts, suggesting that melanin alone may not fully account for EP loss in aging BALB/cJ mice [89]. These findings are consistent with reports of accelerated age-related EP decline in the albino strains of rats (Fischer 344/NHsd) [117], further albino mouse strains (NOD.NON-H2nb1) [118] and albino guinea pigs [119]; however, these models lack appropriate pigmented controls. Importantly, the lack of melanin does not appear to affect the development of the inner ear in any of these models. In addition to albinism, NOD.NON-H2nb1 mice show a broader pathology including hair cell and neuronal loss, reduced strial thickness, as well as vascular atrophy in the SV [118], which is further discussed below. Interestingly, there are also indications that melanin is not essential for maintaining normal auditory function. Transgenic YRT2 mice, carrying a functional copy of the Tyr locus and indistinguishable from wild-type pigmented animals, as well as transgenic TyrTH mice, which can only generate the melanin precursor L-DOPA, but not melanin, maintained normal auditory thresholds as they aged, while their non-transgenic albino NMRI littermates showed premature HL with age, suggesting that the melanin precursor L-DOPA is sufficient to provide a protective effect against premature deafness [120]. Finally, no correlation between the presence or absence of melanin and the rate of strial degeneration with age was found when comparing the pathology between two albino rat strains (Fischer 344 and Lewis) and two pigmented rat strains (Lewis–Brown Norway F1 and Brown Norway) [121], arguing against a protective role of melanin in age-related HL.

The evidence in favor of melanin’s involvement in the susceptibility to strial pathology comes from experiments examining the susceptibility of albino animals to noise-induced HL. The albino compared to pigmented guinea pigs showed an increased susceptibility to HL due to noise overexposure [122]. Moreover, in normally pigmented chinchillas, the hyperpigmentation of the SV, due to melanin granules in the intermediate cells and, less frequently, the marginal and basal cells, has been observed after noise overexposure [123].

In humans, ethnicity is associated with the development of HL, with adult African American individuals showing a lower prevalence of HL than adult Caucasian individuals [124,125]. A correlation also exists between the ethnicity and pigmentation in the SV. African American SV samples contain significantly more pigmentation than Caucasian specimens, both as children and as adults [126,127]. Consistent with this, several indicators of melanin content, such as eye color and skin sun-sensitivity, are correlated with susceptibility to temporary auditory threshold shift after noise overexposure [128,129,130]. In aging humans, as well as in C57BL/6 and CBA/CaJ mice, strial melanin content significantly increases with age. It is currently unclear whether this increase with age is related to the potential protective role of melanin or whether it is simply a metabolic by-product [126,127,131].

### 3.3. Tissue-Resident Macrophages and Inflammatory Responses

In this section, the role of inflammation and tissue-resident macrophages in the SV are discussed. Tissue-resident macrophages are sentinel immune cells that are involved in initiating inflammatory responses, clearing debris and aggregated pigment granules, maintaining a homeostatic tissue environment, and supporting angiogenesis [132,133]. Tissue-resident macrophages are part of the blood–labyrinth barrier discussed in Section 3.3. Perivascular macrophages (PVMs) are a unique subset of tissue-resident macrophages associated with blood vessels [134]. A number of studies have started to use the term “perivascular macrophage-like melanocytes” (PVM/Ms) for a hybrid cell type containing both macrophage as well as melanocyte markers [73,135,136,137]. Other studies, however, argue against the existence of this hybrid cell type [133,138].

PVMs are integrated into the cochlear vessels and maintain the integrity of the barrier between the blood and the intrastrial space by affecting the expression of tight- and adherens-junction proteins [135]. Their exact role in the regulation of barrier integrity is not yet clear. One study demonstrated that the absence of PVMs resulted in the increased capillary permeability of cultured endothelial cell monolayers to fluorescein isothiocyanate (FITC)-dextran, the increased capillary leakage of various tracers, and a reduction in the EP [135]. In contrast, however, it has also been reported that PVM depletion does not alter the baseline permeability of capillaries for fluorescein, and, in fact, activated PVMs may play a role in the breakdown of the BLB after systemic exposure to lipopolysaccharide [138]. Since different tracers were used to assess the leakage of intact capillaries, selective permeability for different tracers could explain the discrepancy in permeability between the two studies. More recently, an increase in cochlear macrophages was found during inflammation induced by lipopolysaccharide [139]. The excess proliferation and activation of PVMs could result in dysfunctional BLB maintenance. Investigating the numbers and activation states of PVMs under different circumstances could provide insight into these potentially pathological mechanisms.

Recently, a study by Lang et al. [140] revealed that in mice, early pathological changes in the SV are associated with increased macrophage activation and “inflammaging”, which is characterized by chronic low-grade inflammation with a dysregulation of immune cell activity. The age dependent increase in macrophage activation was correlated with an increase in cochlear auditory brainstem response (ABR) thresholds. Strial thickness was, however, not correlated with age or thresholds, highlighting once more that strial thickness is not a reliable indicator of strial function [140]. These results indicate that the dysregulation of the immune system and macrophage dysfunction could be early indicators of age-related strial pathology. Macrophage activation has been demonstrated before in the human cochlea [141,142]. As in mice, in human strial samples the number of activated macrophages also increased significantly with age while strial thickness did not differ significantly [140].

### 3.4. Vascular Atrophy

This section synthesizes numerous findings from animal models linking the atrophy of the strial vasculature to the age-related decline in cochlear function. In C57BL/6 mice aged 6–12 months, corrosion casts, which create detailed three-dimensional replicas of blood vessels, revealed the degeneration of the vasculature in the SV, especially in basal turns, where the capillary diameter was significantly reduced [143]. In C57BL/6 and BALB/cJ mice, the age-related thickening of the basement membrane and reduced capillary diameter were observed, both of which may contribute to chronic ischemia within the cochlea [89]. Surprisingly, however, there was no correlation between the EP and either the basement membrane thickness or capillary diameter in either strain [89]. In fact, in old BALB/cJ mice, low EPs tended to be associated with larger capillaries. Thus, the dependency of the EP on physical features of the basement membrane and the strial vasculature is not straightforward.

Gerbils also show age-related changes in strial vasculature. As in mice, the basement membrane thickness of SV capillaries was increased in gerbils aged 33 months and older [144]. With advancing age, the loss of capillaries in regions of the SV progressed from the extreme apical and basal ends and into the middle regions of the cochlea [145]. Small areas of the SV with few or no capillaries were, in fact, already observed at the extreme apical and basal ends of the SV by 5–9 months of age. By 33 months of age, the regions of the SV with normal vasculature were found only in the middle and upper basal turns, with the rest of the SV showing a loss of capillaries and, in some cases, the atrophy of the marginal cells as well. These findings suggest that changes in the strial microvasculature begin before the onset of HL and may initiate the degeneration of other parts of the SV, including the marginal cells [145]. In a subsequent study, the decline in the strial capillary area in quiet-aged gerbils was quantified, showing a significant reduction in the individual capillary area of 8–29% at 2, 20, and 40 kHz in gerbils aged 33–40 months [146]. The functional consequences of the loss of strial capillaries are, nevertheless, not clear. Although blood flow within the cochlea, and especially SV, is reduced in 2–3-year-old gerbils, reduced blood flow is not clearly related to the loss of strial capillaries but possibly results from decreased perfusion pressure or increased vascular resistance instead [147].

As in mice, in gerbils the link between age-related changes in strial vasculature and the EP is not straightforward and may be difficult to identify in cross-sections given the patchy nature of strial degeneration [146]. Vascular abnormalities assessed in whole-mount preparations were associated with a decrease in EP in gerbils aged in quiet for 36–39 months [148]. Areas with atrophied capillaries were mainly found in the apex, lower base and hook region of the cochlea. The total area in which the capillaries remained normal ranged from 19 to 87% [148]. The EP also varied greatly, ranging from 23 to 83 mV. A significant correlation was found between the area with normal vasculature and the mean EP (measured at several locations) [148].

Recent work has also uncovered a link between HL caused by cytomegalovirus (CMV) infection and a decreased EP in BALB/c and C57BL/6 mice [149]. Congenital CMV infection is the most common congenital viral infection and often results in sensorineural HL in infants [150]. In BALB/c mice, CMV infection is associated with capillary network damage in the SV and elevated ABR thresholds [151]. CMV pathology is, however, not limited to the SV, and even within the SV it remains unclear whether capillary damage is the initial step in strial degeneration. Although capillary damage and insufficient oxygen supply could lead to disrupted ion transport and decreased EP, ion transport may be disrupted before vascular damage occurs [149]. It has also been suggested that damage to the SV could be caused by an inflammatory response in the inner ear [152]. The role of CMV infection in HL has been reviewed more extensively elsewhere [16,17,18].

## 4. Conclusions

This review summarizes a large body of research identifying correlations between age-related changes in the structure and function of the SV with disruptions in ion transport, altered pigmentation, inflammatory responses, and vascular abnormalities. These insights underscore the crucial role of the SV in cochlear function and its contribution to age-related HL and, moreover, motivate continued research to identify the mechanisms linking age-related changes in the SV and age-related HL. Identifying these mechanisms is crucial for developing an array of individualized strategies to treat age-related HL and requires increased attention as part of ongoing research endeavors, which are increasingly focused on the contribution of the sensorineural structures within the cochlea.

Future research should explore the complex interplay between the SV, EP generation, and its impact over time on age-related HL. To this end, experiments should recognize that, as highlighted in this review, the SV comprises distinct cell types that operate in an interdependent and synergistic manner and, thus, requires a diverse toolkit that permits investigation from cells to tissue. Single-cell approaches include electrophysiological approaches capable of probing ion transport mechanisms in living cells to transcriptomic and proteomic approaches capable of investigating, in high throughput, age-related changes in gene and protein expression within single, identified cell types. At the tissue level, advanced imaging techniques, such as high-resolution microscopy and imaging modalities that capture dynamic blood flow, will be necessary to visualize and analyze gradual age-related changes in the size and blood flow within the SV. These methods should be applied to existing animal models and, in addition, further explore strain differences in mice, better utilize existing transgenic models, and potentially employ CRISPR-Cas technology to generate new transgenic animal, and especially gerbil, models.

Ultimately, these approaches and others, in combination with clinical research, will delineate the various mechanisms including genetic and epigenetic factors and environmental stressors that determine and influence the susceptibility of the SV to age-related changes and identify strategies for intervention and prevention. Achieving this ambitious goal necessitates ongoing interdisciplinary collaboration, a longstanding strength of auditory research.

## Data Availability

No new data were created or analyzed in this study. Data sharing is not applicable to this article.
